# Hydrogenotrophic Methanogenesis and Autotrophic Growth of *Methanosarcina thermophila*

**DOI:** 10.1155/2018/4712608

**Published:** 2018-07-17

**Authors:** Nina Lackner, Anna Hintersonnleitner, Andreas Otto Wagner, Paul Illmer

**Affiliations:** Department of Microbiology, Universität Innsbruck, Technikerstraße 25d, 6020 Innsbruck, Austria

## Abstract

Although Methanosarcinales are versatile concerning their methanogenic substrates, the ability of *Methanosarcina thermophila* to use carbon dioxide (CO_2_) for catabolic and anabolic metabolism was not proven until now. Here, we show that *M. thermophila* used CO_2_ to perform hydrogenotrophic methanogenesis in the presence as well as in the absence of methanol. During incubation with hydrogen, the methanogen utilized the substrates methanol and CO_2_ consecutively, resulting in a biphasic methane production. Growth exclusively from CO_2_ occurred slowly but reproducibly with concomitant production of biomass, verified by DNA quantification. Besides verification through multiple transfers into fresh medium, the identity of the culture was confirmed by 16s RNA sequencing, and the incorporation of carbon atoms from ^13^CO_2_ into ^13^CH_4_ molecules was measured to validate the obtained data. New insights into the physiology of *M. thermophila* can serve as reference for genomic analyses to link genes with metabolic features in uncultured organisms.

## 1. Introduction

Biogenic methane (CH_4_) is produced by methanogenic archaea, using three main substrates: acetate, CO_2_, and substances containing a methyl group [1] (Table 1). Among all methanogenic archaea, only the order Methanosarcinales includes members able to metabolize all three substrates [1]. Acetoclastic methanogenesis is exclusively performed by the genera *Methanosarcina* and *Methanosaeta*, both members of the Methanosarcinales, which differ in their substrate specificity and their affinity to acetate [1, 2]. Methylotrophic methanogenesis can be hydrogen-dependent or hydrogen-independent and is limited to Methanosarcinales, Methanomassiliicoccales, and one species of Methanobacteriales [1, 3]. Furthermore, genome analyses suggest hydrogen-dependent methylotrophic methanogens in the new phylum Verstraetearcheota [4]. Hydrogen-dependent species use hydrogen (H_2_) to reduce the methyl group to CH_4_ [1, 5]. Hydrogen-independent methanogenesis involves the reduction of methyl groups with electrons deriving from the oxidation of further methyl groups, so that for each three CH_4_ molecules, one molecule of CO_2_ is produced [1, 5].

Contrary to the two preceding pathways, hydrogenotrophic methanogenesis, the reduction of CO_2_ with H_2_ to CH_4_, can be performed by nearly all methanogens. Among them, obligate CO_2_-reducing methanogens and microorganisms able to use a broad range of substrates can be distinguished. They differ in some of the involved enzymes and the mode of energy conservation [6]. Organisms thought to be unable to perform hydrogenotrophic methanogenesis are found solely within the Methanosarcinales. It was shown for instance that the mesophilic methanogen *Methanosarcina acetivorans* is unable to use CO_2_ for methanogenesis [1].

The organism *Methanosarcina thermophila* was firstly described under the name TM-1 by Zinder and Mah in 1979 [7]. It was isolated from a thermophilic anaerobic sludge digester and is able to metabolize acetate, methanol, methylamine, and trimethylamine [7]. In the last few years, *M. thermophila* was repeatedly detected in various biogas fermenters with molecular methods, which indicates that it might play a central role in active communities of anaerobic digesters [8–10]. The methanogen is thought to be crucial to overcome process disturbances due to high acetate levels in biogas reactors [11, 12] and to be outstandingly resilient encountering changing temperatures during anaerobic digestion [13]. The observations in literature about the ability of *M. thermophila* to use CO_2_ as a methanogenic substrate and a carbon source range from no methanogenesis or growth [7, 14] to weak growth [15] on CO_2_, but no concrete data is published concerning this topic.

In the past years, sequencing approaches revealed new distinct groups of archaea that were classified as potential methanogens due to specific genes linked to methanogenesis [4, 16, 17]. The physiological characterization of cultivable methanogens is crucial to validate the correlation between molecular data and functional traits. Therefore, we investigated the consumption of H_2_ and CO_2_ by *M. thermophila* cultivated either with methanol as co-substrate or without organic substrates. Further, we determined the rate of CH_4_ production, acetate excretion, and DNA yield during the autotrophic incubation of *M. thermophila*.

## 2. Material and **Methods**

### 2.1. Media and Incubation Conditions

The mineral medium contained per liter 0.35 g K₂HPO₄, 0.23 g KH₂PO₄, 0.244 g MgSO₄, 0.25 g CaCl₂∗2H₂O, 2.25 g NaCl, 0.002 g FeSO₄∗7 H₂O, 2.49 g NH₄Cl, 1 mL resazurine solution (0.115% *w*/*v*) as redox indicator, 1 mL trace mineral solution (SL-10 DSMZ medium 320), 20 mL NaHCO₃ solution (10% *w*/*v*), and 975 mL distilled water. The medium was flushed with a N_2_/CO_2_ mixture (70 : 30) and simultaneously cooled down to approximately 5°C to enable additional CO_2_ to dissolve. After the pH was adjusted to 6.8, 50 mL of medium was anaerobically aliquoted in 250 mL serum bottles, which were flushed with either a N_2_/CO_2_ (70 : 30) or a H_2_/CO_2_ (80 : 20) gas mixture to guarantee anaerobic conditions. Subsequently, the bottles were sealed and autoclaved. The sterile medium was amended with 0.2 mL Na_2_S∗9 H_2_O solution (23.1% *w*/*v*), 0.2 mL cysteine-HCl solution (7.5% *w*/*v*), and 0.5 mL vitamin solution (VL-141 DSMZ) per bottle. Due to earlier protocols, 2 mL erythromycin solution (0.1% *w*/*v*) was added per bottle to avoid bacterial infections right before the inoculation. This precautional measure proved to be unnecessary, as no contaminations of the culture appeared, when it was inoculated in a rich medium containing no erythromycin at the end of the investigation. Furthermore, 0.25 mL pure methanol were amended if necessary. To raise the partial pressure of the substrate gases, headspaces were upgraded initially with 100 mL extra filter sterilized gas. The Na_2_S and the cysteine-HCl solutions were autoclaved; the vitamin solution, the erythromycin solution, and the methanol were filter sterilized. The samples were inoculated with *Methanosarcina thermophila* TM-1 (DSM strain 1825, obtained from DSMZ-German Collection of Microorganisms and Cell Cultures, Germany) via a syringe and incubated at 50°C ± 0.5°C and 70 rpm in a closed batch system.

### 2.2. Gas and Chemical Analysis

To quantify gas amounts, the overpressure in the headspaces of the bottles was measured with a digital precision monometer (GDH 200-13, Greisinger electronic, Germany) and normalized with the ambient pressure (data from ZAMG (Zentralanstalt für Meterologie und Geodynamik, Austria)). The gas composition was determined with a Shimadzu GC2010 as described in [18], using a TCD (thermal conductivity detector). The samples were taken and immediately injected with 1 mL syringes. The pH value was monitored to ensure stable incubation conditions. It was measured with a glass electrode and was invariable in all experiments. For the analysis of acetate concentrations, 1 mL samples were centrifuged for 10 min at 20.000 ×g to remove solid components. The supernatants were filtrated through a 0.2 *μ*m RC (Phenomenex, Germany) filter and analyzed via HPLC on a Shimadzu Prominence system as described before [19]. To observe the incorporation of carbon atoms from CO_2_ molecules into CH_4_ molecules, 10 mL ^13^CO_2_ (36% (*v*/*v*), diluted in carbon-free air (Messer, Austria)), was added to the headspace of the serum bottles. The proportion of ^13^C in CO_2_ and CH_4_ gas was determined with a Picarro G2201-i Analyzer (USA).

### 2.3. DNA-Based Analysis

To quantify the dsDNA content in the culture fluid, genomic DNA was extracted from the pellet of 1 mL culture fluid using a NucleoSpin® Soil Kit (MACHERY-NAGEL, Germany). Extraction was performed according to the manufacturer protocol, using SL1 in the first lysis step. The DNA content in the extracts was measured with a Quantus™ Fluorometer (Promega, USA, Cat number E6150). To ensure the identity of the culture and to exclude an infection with another hydrogenotrophic microorganism, DNA from a well growing sample was extracted at day 21. Genomic DNA was amplified by PCR, using the archaeal primers 109f [20] and 1492r [21]. The PCR mix contained per reaction volume of 50 *μ*L: 19.4 μL PCR grade water, 26.4 μL Red Taq DNA Polymerase 2x Master Mix (VWR, USA, Cat. number 733-2547), 1.1 μL of each primer, and 2 μL template. The reaction was executed in a FlexCycler (Analytik Jena, Germany) with 10 min at 95°C for initial denaturation, followed by 35 cycles of 30 s at 95°C, 30 s at 52°C, and 45 s at 72°C. The PCR product was sequenced by Eurofins Genomics (Germany), and the resulting nucleotide sequences were analyzed with NCBI BLAST.

### 2.4. Statistics

The statistical analyses were performed using STATISTICA 12 (StatSoft®). After testing the data for normality and homogeneity of variance, significant differences between groups were calculated by one-way or multivariate ANOVA (analysis of variance). To assess relationships between variables, a Pearson correlation was used. The alpha level used throughout was 0.05 for significant and 0.01 for highly significant results.

## 3. Results

### 3.1. Growth on Methanol and CO_2_

**Figure 1:**
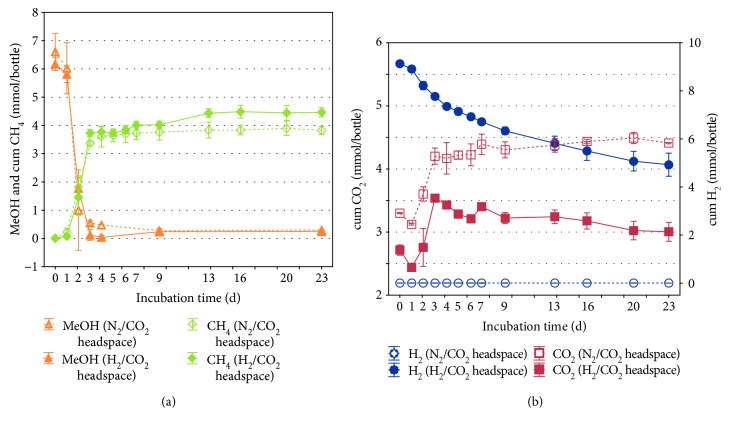
Decreasing methanol (MeOH) concentration (a) in the medium, cumulative CH_4_ (a), H_2_ (b), and CO_2_ (b) in the headspace of a *Methanosarcina thermophila* culture with an initially either N_2_/CO_2_- or H_2_/CO_2_-containing headspace within 23 days of incubation (means; whiskers: standard deviation).

In a first approach, *Methanosarcina thermophila* was grown on a mineral medium containing methanol and H_2_/CO_2_ in the headspace (Figure 1). The headspace of two inoculated samples was replaced by a sterile N_2_/CO_2_ mixture, serving as H_2_-free controls to quantify the gas fluxes generated during the degradation of methanol (Figure 1). A not inoculated negative control, containing H_2_/CO_2_ in the headspace (data not shown), resulted in no CH_4_ production, and the H_2_ and CO_2_ contents stayed unchanged over the whole incubation period of 23 days. The presence of H_2_ in the bottles had a positive effect on the cumulative CH_4_ production and a negative effect on the net CO_2_ production after 23 days. To quantify gas fluxes occurring separately from the methanol degradation, the net gas turnover in the H_2_-free controls was subtracted from the net gas turnover in the H_2_-containing bottles. Referring to Figure 1, the results showed that H_2_ variants consumed 4.21 mmol H_2_ and 0.82 mmol CO_2_ as well as produced 0.66 mmol CH_4_ more than the H_2_-free controls.

### 3.2. Growth on H_2_/CO_2_

In a next step, a mineral medium, containing solely CO_2_ as carbon source and H_2_ as electron acceptor, was inoculated with 0.1 mL sediment of an active culture of *M. thermophila*, grown on a methanol-acetate medium. The small inoculation volume was chosen to prevent the transfer of potential organic carbon sources. In the first generation of such setup, three of nine samples produced CH_4_ during 38 days of incubation (data not shown). One of the samples actively producing CH_4_ of the first generation was frozen and subsequently utilized to inoculate (0.1 mL) the second generation of *M. thermophila* grown on H_2_/CO_2_. In this trial, three out of five samples produced between 1.4 and 1.7 mmol CH_4_ within 56 days of incubation, with lag phases ranging from zero to 21 days. The other two samples and the negative controls, bottles containing either no inoculum or no H_2_, did not yield any CH_4_. The theoretical potential CH_4_ production (disregarding anabolism), calculated according to the available CO_2_ and H_2_ content at the beginning of the incubation, would have been 2.50 and 2.33 mmol per bottle, respectively (Table 1). Therefore, the actual measured CH_4_ production could mathematically derive from the reduction of CO_2_ and accounts for approximately 65% of the potential CH_4_ production. The sequencing results of an aliquot of culture fluid from day 21 showed 99.69% identity of the sample with the ordered *Methanosarcina thermophila* strain DSM 1825 (NCBI accession number: AB973357.1).

**Figure 2:**
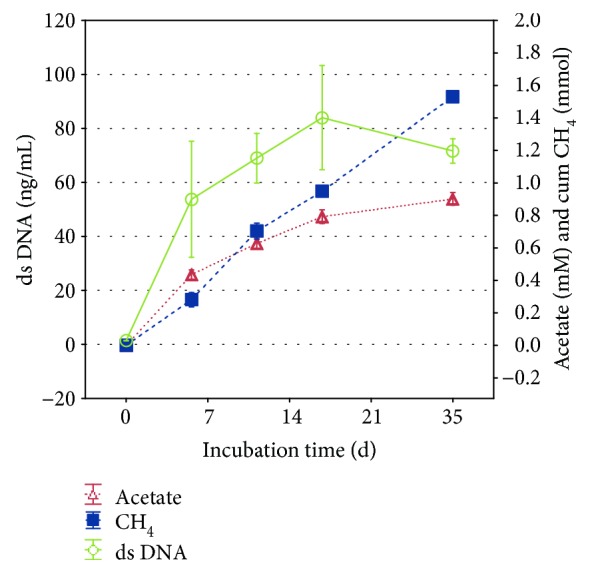
Acetate and DNA content in the culture fluid and cumulative CH_4_ production by *Methanosarcina thermophila* in an organic carbon-free medium with a H_2_/CO_2_ headspace (means; whiskers: standard deviation).

From the next experiment, all incubation bottles were inoculated with 1 mL of an active CO_2_ culture to ensure a higher rate of successful cultivations than achieved with 0.1 mL transfer volume. Indeed, in generation three, all six samples showed visible growth. In three of six parallels, *M. thermophila* was incubated in a medium lacking cysteine and erythromycin to detect possible CH_4_ production, resulting from the utilization of those two medium components as methanogenic substrate. The presence or lack of cysteine and erythromycin had no significant effect on the cumulative CH_4_ production or the cumulative CO_2_ and H_2_ consumption until the end of the incubation (multivariate ANOVA: *p* = 0.58). The average CH_4_ yield was 1.53 ± 0.03 mmol, the average H_2_ consumption 5.53 ± 0.25 mmol, and the average CO_2_ consumption −0.55 ± 0.14 mmol in all six bottles after 35 days. At this point of the incubation, the pressure in the bottles was already negative, as for every produced molecule of CH_4_ five substrate molecules are consumed (Table 1). For this reason, gas measurements at later time points were less trustworthy and therefore not taken into account for data analysis, although CH_4_ concentration in the headspace continued to increase. Hydrogenotrophic methanogenesis in three parallels of the third generation (with erythromycin and cysteine) was further characterized concerning DNA content and concentration of acetate in the medium (Figure 2). The concentration of acetate reached up to 0.90 mM, which is the equivalent of 0.05 mmol/bottle.

### 3.3. Carbon Flow and Methanogenic Performance

To validate whether the carbon of the produced CH_4_ molecules derived from CO_2_ molecules, ^13^C-labelled CO_2_ was added to two of three parallels of the fourth generation. The addition of 10 mL CO_2_ with 37% ^13^C resulted in an average ^13^CO_2_ concentration of 5.22% in the headspace of the two samples. After 3 weeks and an average CH_4_ production of 0.75 ± 0.12 mmol, the ^13^C content of the produced CH_4_ was approximately 3.62% and thus in the same range as the ^13^C content of the remaining CO_2_ (approximately 3.46%) in the labeled bottles. The ^13^C proportions of CH_4_ (1.07%) and CO_2_ (1.02%) in the bottle without labelled CO_2_ were, however, distinctively lower and within the natural range. Thus, it can be concluded that the labeled carbon atoms were transferred from the CO_2_ pool to the CH_4_ pool.

**Figure 3:**
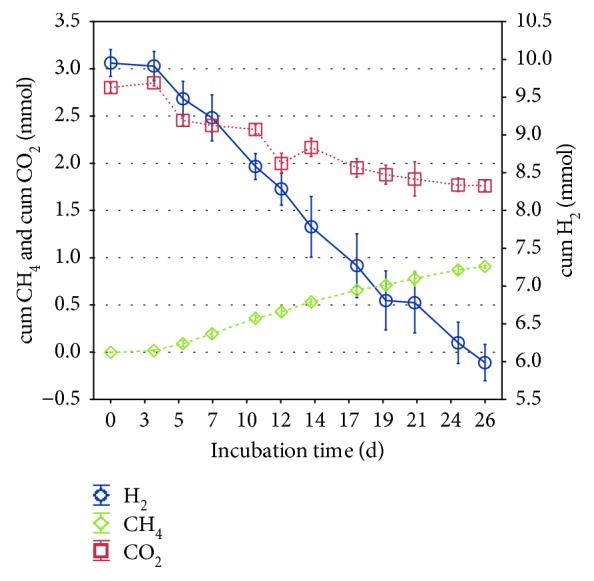
Cumulative CH_4_ production, cumulative H_2_ consumption, and cumulative CO_2_ consumption by *Methanosarcina thermophila* growing in a medium containing only CO_2_ as methanogenic substrate (means; whiskers: standard deviation).

**Figure 4:**
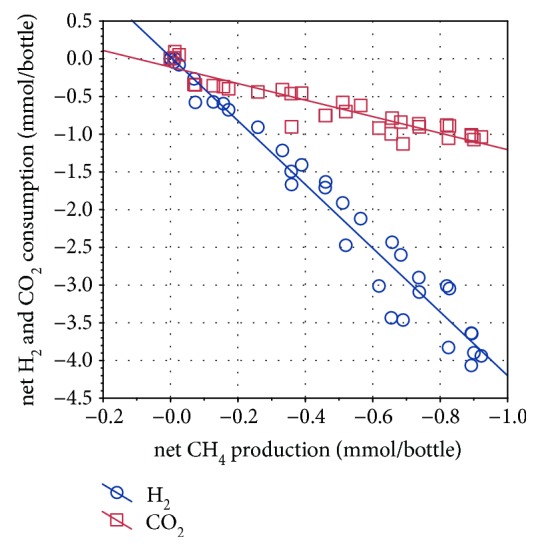
Linear correlation between CH_4_ production and CO_2_/H_2_ consumption by *Methanosarcina thermophila* during 26 days of incubation (Pearson correlation: CO_2_: *p* ≤ 0.01, *R*^2^ = 0.88; H_2_: *p* ≤ 0.01, *R*^2^ = 0.96).

During the fifth generation, the sampling intervals of three parallels were shortened to quantify the rate of hydrogenotrophic methanogenesis performed by *M. thermophila*. From day 3 onwards, CH_4_ production showed a rather linear (*R*^2^ = 0.97, *p* < 0.01) than exponential pattern, with an average rate of CH_4_ production of 0.04 mmol/day (0.11 mmol/day/L initial H_2_/CO_2_) (Figure 3). Further, there was a strong linear correlation between the production of CH_4_ and the consumption of H_2_ and CO_2_, respectively (Figure 4). To complete the investigations, autotrophically grown cells were microscopically compared with cells grown on methanol and acetate. As also confirmed by sequencing data, there were no signs for contaminations in the culture grown on CO_2_. The comparison of heterotrophically and autotrophically cultivated organisms showed decreased fluorescence in CO_2_ cultures, indicating a lower level of the molecule F_420_ and therefore a lower methanogenic activity in those cells, corresponding to the different CH_4_ production rates on methanol and H_2_/CO_2_ (Figure 1).

## 4. Discussion

The present study on autotrophic growth by *Methanosarcina thermophila* started with the investigation of CO_2_ and H_2_ as co-substrates of methanol. The collected data from gas measurements showed a biphasic CH_4_ production of *M. thermophila*, with a second lag phase, occurring during the shift from consumption of the preferred substrate methanol to consumption of CO_2_ (Figure 1). Interestingly, previous studies investigating *Methanosarcina bakeri* strain 227 and strain MS by Ferguson and Mah [22] as well as Hutten et al. [23] did not observe a biphasic growth pattern. In the present study, the observed CO_2_ production, during the degradation of methanol, was consistent with the stoichiometry of the hydrogen-independent methylotrophic methanogenesis, with every fourth methanol molecule being oxidized to CO_2_ [1]. This pathway of methanol degradation was also suggested for the genus *Methanosarcina* by Zinder [24]. After the depletion of methanol, CH_4_ production continued, although slower, and was accompanied by decreasing H_2_ and CO_2_ levels. Therefore, it could be shown that *M. thermophila* is able to perform hydrogenotrophic methanogenesis in a methanol-CO_2_ medium (Figure 1). Reduction of CO_2_ in the presence of methanol was already uniformly observed by Zinder and Mah [7] as well as Mladenovska and Ahring [14]. Their findings, however, deviate from each other concerning the CO_2_ consumption after the depletion of methanol. Zinder and Mah [7] stated that metabolism of H_2_ stopped as soon as methanol was depleted, whereas Mladenovska and Ahring [14] found ongoing methanogenesis after methanol was exhausted. As mixotrophically grown cells transferred into a new H_2_/CO_2_ medium did not show any growth or CH_4_ production during their experiments, Mladenovska and Ahring [14] further stated the hypothesis that methanol seems to be critical for cell formation, which was clearly not true for the culture used in the present experiments.

The ability or inability of *M. thermophila* to produce CH_4_ from CO_2_ as a sole methanogenic substrate is mentioned in various articles, but there are only two publications in which the topic was experimentally investigated. Zinder and Mah [7] did not succeed to grow *M. thermophila* autotrophically during their initial isolation and characterization of the organism in 1979 and stated further that they found no clear explanation for this fact. In 1985, Zinder et al. [15] stated that growth of *M. thermophila* “may occur slowly on H_2_-CO_2_,” but the corresponding data were not published and only distributed to other authors via personal communication [25]. Therefore, the present study was conducted to provide the first concrete data on the autotrophic growth of *M. thermophila* (Figure 2). Several measures were taken to assure that the CH_4_ actually was produced by *M. thermophila* and derived from CO_2_. The possibility of CH_4_ production from organic carbon in the inoculation material was eliminated by multiple transfers of small volumes into fresh medium. The carbon-containing medium components, erythromycin and cysteine, were also excluded as methanogenic substrates. Further, the identity and purity of the methanogen culture were confirmed via microscopy and DNA sequencing. Minor differences in the sequences are due to ambiguities in the sequencing.

During the incubation of *M. thermophila* in the absence of organic methanogenic substrates, CH_4_ production as well as H_2_ and CO_2_ consumption largely corresponded to the stoichiometric model in which four molecules of H_2_ and one molecule of CO_2_ are used to produce one molecule of CH_4_ (Figure 4). Furthermore, the actual transfer of labeled carbon atoms from the CO_2_ to the CH_4_ pool via hydrogenotrophic methanogenesis could be shown. The fact that *M. thermophila* produced and excreted acetate, although it was grown under oligotrophic conditions and acetate being the preferred substrate compared with H_2_/CO_2_, was unexpected (Figure 2). Similar observations were made, however, by Westermann et al. [26], demonstrating that *Methanosarcina barkeri* released acetate up to millimolar concentrations into the surrounding media, as did *Methanosarcina mazei*, although in smaller quantities. A possible explanation for these findings is that acetate is produced in the course of assimilation of CO_2_ into cell carbon via intermediates including activated acetic acid or acetyl coenzyme A [27] and subsequently leaks the cell by passive diffusion [28]. The reuptake of lost acetate is limited by the minimum threshold for acetate utilization by *Methanosarcina* spp., which is known to be in the range of 0.2 to 1.2 mM [29]. This could explain the continuously increasing acetate concentration during the autotrophic methanogenesis by *M. thermophila* and may provide an indication that the organism is integrating carbon from CO_2_ into the biomass. Apart from this, the present data further supports the evidence that *M. thermophila* is not only producing CH_4_ from CO_2_ and H_2_ but is also generating biomass autotrophically. As the specific growth morphology of the Methanosarcinales prevented the direct quantification of the cell number, the production of biomass, although at a low level, was determined by quantifying the DNA content in the culture fluid (Figure 2). Contrary to the findings of Zinder and Mah [7] for methanogenesis from acetate and methanol, CH_4_ production from H_2_/CO_2_ was rather linear than exponential and much slower than growth on acetate or methanol. However, linear methane production was also observed for *Methanosarcina bakeri* showing a CH_4_ production rate of 0.23 mmol/day/L initial H_2_/CO_2_ under similar incubation conditions, with the determined rates being twice as high compared with this study [23]. Low methane production rates from H_2_/CO_2_ might have been attributed to the high molar volume of gases limiting substrate addition, the diffusion of gases into the nutrition medium, and the challenging adaptation to a new type of methanogenic substrate. Further, authors investigating hydrogenotrophic methanogenesis by *Methanosarcina* spp. found higher growth rates in complex media than in mineral media [22, 30]. The role of *M. thermophila* as hydrogenotrophic methanogen in biogas production can only be estimated from the obtained data, as the applied H_2_ partial pressure was much higher than in a bioreactor. Most acetogenic reactions require a H_2_ partial pressure below 10^−4^ bar to be thermodynamically favorable [31]. According to Lovley and Ferry [32], *M. thermophila* produced and consumed H_2_ to maintain H_2_ partial pressures between 0.67 and 1.6 mbar during growth on acetate or methanol, indicating that the threshold for hydrogen uptake is rather low. Furthermore, Maestrojuan and Boone found that *Methanosarcina vacuolata* produced only 30–40% of the expected methane in a mineral medium containing H_2_/CO_2_, probably due to decreasing substrate concentrations shifting thermodynamics [30].

## 5. Conclusions

*Methanosarcina thermophila* showed a biphasic CH_4_ production growing mixotrophically on methanol and H_2_/CO_2_, switching from primarily methylotrophic methanogenesis to hydrogenotrophic methanogenesis as soon as methanol was depleted. Furthermore, it could be shown that *M. thermophila* is, contrary to the common opinion, able to perform hydrogenotrophic methanogenesis independently from other methanogenic substrates and to build up biomass autotrophically. Achieved CH_4_ production rates were lower than those commonly found during methanogenesis from the preferred substrates acetate or methanol, but although carbon supply during incubations was restricted by the available volume of the headspace, *M. thermophila* successfully built up visible amounts of biomass. Further, the comprehensive physiological characterization of organisms is the foundation of functional genome analyses. Experimental data on the metabolic abilities of cultured methanogens are crucial to draw conclusions on the metabolic capabilities of uncultured archaea. We hope that the present study will help future investigations to refine this linkage.

## Figures and Tables

**Table 1:** Methanogenic pathways and free energies of the respective central reactions under standard conditions modified from Liu and Whitman [1].

Methanogenic pathway	Reaction of CH_4_ formation	*∆G* ^0′^ (kJ/mol)
Acetoclastic methanogenesis	CH_3_COO^−^ + H^+^ → CH_4_ + CO_2_	−33
Hydrogen-independent methylotrophic methanogenesis	4 CH_3_OH → 3 CH_4_ + CO_2_+ 2 H_2_O	−105
Hydrogen-dependent methylotrophic methanogenesis	CH_3_OH + H_2_ → CH_4_ + H_2_O	−113
Hydrogenotrophic methanogenesis	4 H_2_ + CO_2_ → CH_4_+ 2 H_2_O	−135

## Data Availability

The data used to support the findings of this study are available from the corresponding author upon request.
